# Disrupted Post-Transcriptional Regulation of Gene Expression as a Hallmark of Fatty Liver Progression

**DOI:** 10.3390/ijms252011054

**Published:** 2024-10-15

**Authors:** Shohei Takaoka, Marcos E. Jaso-Vera, Xiangbo Ruan

**Affiliations:** 1 Division of Endocrinology, Diabetes and Metabolism, Johns Hopkins University School of Medicine, Baltimore, MD 21205, USA; stakaok1@jhmi.edu (S.T.); mjasove1@jhmi.edu (M.E.J.-V.); 2 Institute for Fundamental Biomedical Research, Johns Hopkins All Children’s Hospital, St. Petersburg, FL 33701, USA

**Keywords:** post-transcriptional regulation, non-sense mediated decay, RNA-seq, fatty liver diseases

## Abstract

It is known that both transcriptional and post-transcriptional mechanisms control messenger RNA (mRNA) levels. Compared to transcriptional regulations, our understanding of how post-transcriptional regulations adapt during fatty liver progression at the whole-transcriptome level is unclear. While traditional RNA-seq analysis uses only reads mapped to exons to determine gene expression, recent studies support the idea that intron-mapped reads can be reliably used to estimate gene transcription. In this study, we analyzed differential gene expression at both the exon and intron levels using two liver RNA-seq datasets from mice that were fed a high-fat diet for seven weeks (mild fatty liver) or thirty weeks (severe fatty liver). We found that the correlation between gene transcription and mature mRNA levels was much lower in mice with mild fatty liver as compared with mice with severe fatty liver. This result indicates broad post-transcriptional regulations for early fatty liver and such regulations are compromised for severe fatty liver. Specifically, gene ontology analysis revealed that genes involved in synapse organization and cell adhesion were transcriptionally upregulated, while their mature mRNAs were unaffected in mild fatty liver. Further characterization of post-transcriptionally suppressed genes in early fatty liver revealed that their mRNAs harbor a significantly longer 3′ UTR, one of the major features that may subject RNA transcripts to nonsense-mediated RNA decay (NMD). We further show that the expression of representative genes that were post-transcriptionally suppressed were upregulated in mice with a hepatocyte-specific defect of NMD. Finally, we provide data supporting a time-dependent decrease in NMD activity in the liver of a diet-induced metabolic-dysfunction-associated fatty liver disease mouse model. In summary, our study supports the conclusion that NMD is essential in preventing unwanted/harmful gene expression at the early stage of fatty liver and such a mechanism is lost due to decreased NMD activity in mice with severe fatty liver.

## 1. Introduction

Overnutrition-associated obesity increases all forms of cardiometabolic diseases, including hyperlipidemia, diabetes and fatty liver diseases. In response to overnutrition, tissues/organs adapt gene expression/activity to maintain metabolic homeostasis. With the overnutrition-associated stress continuing, tissues/organs exhaust the capacity to adapt gene expression/activity to maintain homeostasis, leading to metabolic diseases [1]. In the past decades, tremendous progress has been made in understanding how genes are transcriptionally regulated in response to major nutrients/hormones. For example, transcription factor SREBP1c controls feeding associated de novo lipogenesis [2], SREBP2 senses cellular cholesterol content to promote cholesterol synthesis [3] and PPAR-α activation promotes fatty acid catabolism [4]. However, at the RNA level, gene expressions are controlled by both transcriptional and post-transcriptional mechanisms. These post-transcriptional mechanisms include splicing and processing of pre-mRNA, transportation of mRNA and nuclear export, translation of mRNA and, ultimately, the degradation of mRNA [5]. Compared to well-studied transcriptional regulation, our understanding of post-transcriptional mechanisms during diet-induced obesity is much less clear [5]. 

The importance of post-transcriptional mechanisms in metabolic diseases is emerging. Recent mouse genetic studies found that knocking out of key components of RNA decay machinery and some RNA-binding proteins (RBPs), and splicing factors, resulted in obesity, abnormal adipose tissue metabolism and liver diseases [6,7,8,9,10,11]. Despite these progresses, how post-transcriptional mechanisms adapt to overnutrition is largely unclear, especially at the whole-transcriptome level due to technical limitations. While ChIP-seq and RNA-seq have been the standard procedures to study transcriptional regulations at tissue/organ levels, most of the established methods for detecting RNA decay in cultured cells, like blocking transcription by actinomycin D or BrU labeling of nascent RNA, are not practical or with limitations in animals [12,13].

The recent advances in high-throughput RNA sequencing have provided huge advantages in defining differential gene expressions at multiple levels. In addition to determining total gene expression levels, extensive RNA-seq analysis can also determine splicing patterns and alternative usage of 3-prime and 5-prime ends [14]. One unique feature of RNA-seq data is that they contain reads that can be mapped to both introns and exons, especially when the RNA-seq library is prepared using total RNA [15]. As such, reads mapped to introns can serve as an estimate of transcription activity and reads mapped to exons can serve as expression of mature mRNA [16]. When analyzing RNA-seq data, by comparing the differential gene expressions at the intron level with those at the exon level, the effects of post-transcriptional regulations can be measured. This strategy was originally proposed by Gaidatzis et al. [17]. and has been applied to determine the role of post-transcriptional regulations in many pathophysiological conditions [18,19]. 

In this study, we established a robust RNA-seq pipeline that determines gene expression at both the intron and exon levels and applied the pipeline to analyze published RNA-seq datasets representing hepatic gene expressions in mice with mild and severe fatty liver [20,21]. Our results revealed broad post-transcriptional regulations in mild fatty liver, and these post-transcriptional regulations were lost in server fatty liver. We further provide evidence that nonsense-mediated RNA decay (NMD) is an important mechanism contributing to post-transcriptional regulations in mild fatty liver.

## 2. Results

### 2.1. Broad Post-Transcriptional Gene Suppression at Early Stage of Fatty Liver

**Figure 1:**
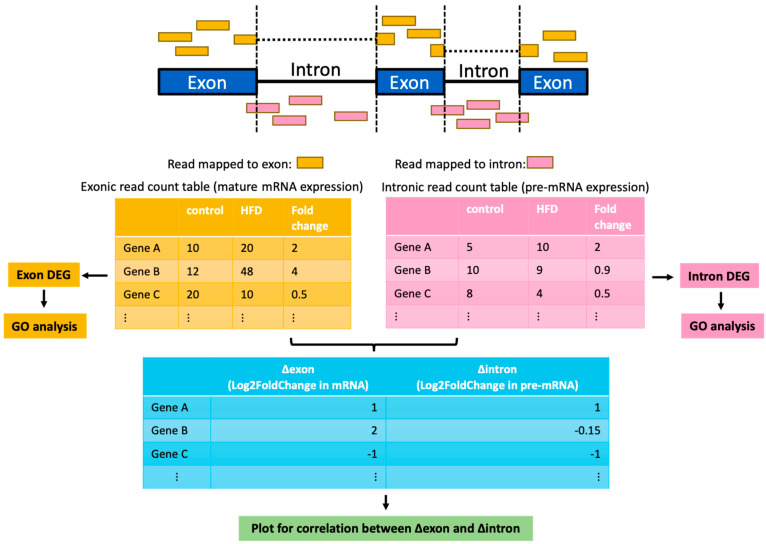
Schematics of the analysis workflow. RNA-seq reads are mapped to the genome and the reads assigned to exon and intron regions are aggregated to calculate the exon and intron counts for each gene, respectively. The obtained count table is used for differential expression analysis by DESeq2 to calculate Log 2Fold change in mature mRNA (∆exon) and Log2FoldChange in pre-mRNA (∆intron). Scatter plots of ∆exon and ∆intron are drawn for each dataset to evaluate how much the change in transcription (∆intron) contributes to the variation in expression in mature mRNA seen in each dataset.

**Figure 2:**
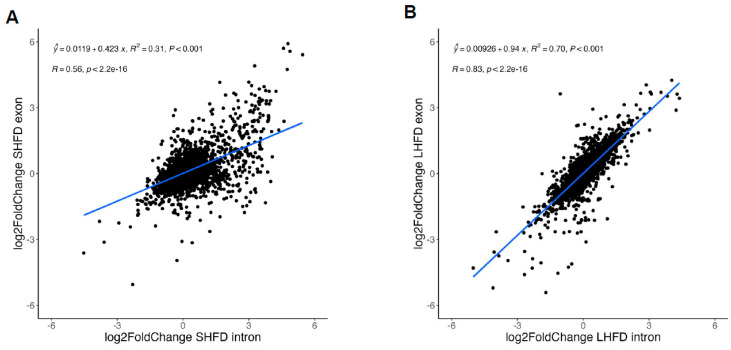
(**A**) The correlations between ∆exon and ∆intron for DEGs with short-term HFD (SHFD). (**B**) The correlations between ∆exon and ∆intron for DEGs with long-term HFD (LHFD). Scatter plots of the results of analyzing each dataset with the analysis pipeline shown in Figure 1.

To systemically define the contribution of post-transcriptional regulations in fatty liver-associated gene expression changes, we searched for published RNA-seq datasets that represent liver gene expression at different stages of fatty liver. Two RNA-seq datasets deposited in NCBI were used: GSE88818 contains RNA-seq results from the liver of mice fed with either a normal chow diet or a high-fat diet (HFD) for seven weeks, representing the stage of mild fatty liver [20]; GSE121340 contains RNA-seq results from the liver of mice fed with either a normal chow diet or an HFD for thirty weeks, thus representing severe fatty liver [21]. The RNA-seq libraries for both datasets were prepared using total RNA (ribosomal RNA depleted), with total reads ranging from 50 to 90 M. To determine differentially expressed genes (DEGs) at both the intron and exon levels, we established the RNA-seq pipeline as shown in (Figure 1). Compared with default RNA-seq analysis, in which RNA-seq reads were only mapped to exon regions, our pipeline map reads to either exon or introns. As there are usually multiple RNA transcripts for a single gene, we only use reads that always map to exons (constitutive exons) or always map to introns (constitutive introns) based on the Ensembl annotation (see Section 4). We detected a total of 469 exon-level and 725 intron-level DEGs for mild fatty liver, and a total of 1975 exon-level and 1577 intron-level DEGs for severe fatty liver (Supplementary Table S1). To globally define the contribution of post-transcriptional regulations, we performed a correlation analysis to determine how intron-level gene expression changes (∆intron) correlate with exon-level gene expression changes (∆exon) (see Section 4). As shown in Figure 2A,B, the R^2^ for this correlation is 0.56 for mild fatty liver and 0.83 for severe fatty liver. This result suggests that gene expression changes at the intron level were less efficiently transferred to gene expression changes at the exon level at the early stage of fatty liver, as compared to what happened in severe fatty liver. It also indicates that broad post-transcriptional regulations at the early stage of fatty liver and gene expressions in severe fatty liver are mainly determined by gene transcription.

### 2.2. Genes Involved in Synapse Organization and Cell Adhesion Were Post-Transcriptionally Suppressed in Mild Fatty Liver

**Figure 3:**
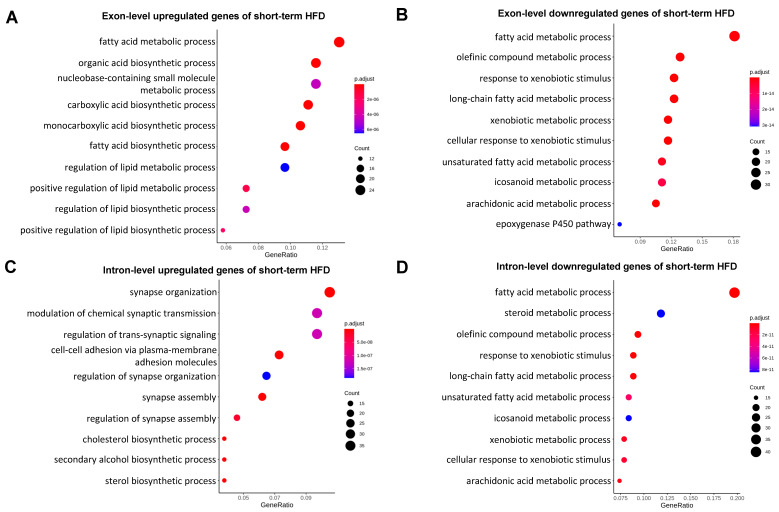
(**A**). GO term analysis for exon-level upregulated genes with short-term HFD, (**B**) GO term analysis for exon-level downregulated genes with short-term HFD, (**C**) GO term analysis for intron-level upregulated genes with short-term HFD, (**D**) GO term analysis for intron-level downregulated genes with short-term HFD.

**Figure 4:**
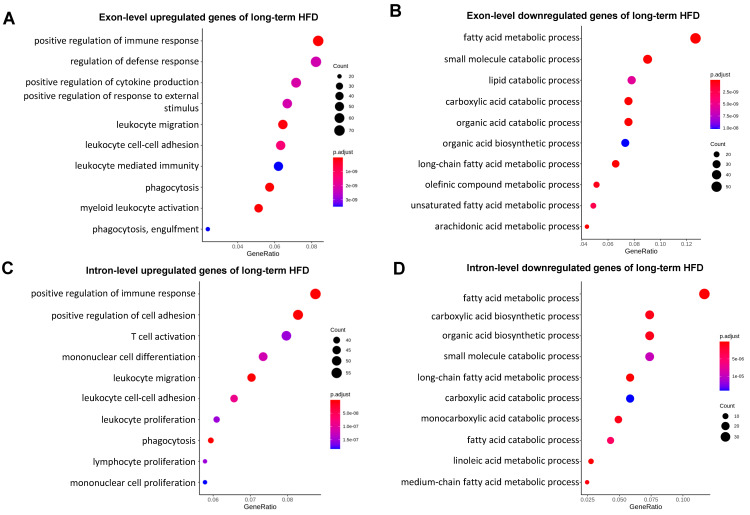
(**A**) GO term analysis for exon-level upregulated genes with long-term HFD, (**B**) GO term analysis for exon-level downregulated genes with long-term HFD, (**C**) GO term analysis for intron-level upregulated genes with long-term HFD, (**D**) GO term analysis for intron-level downregulated genes with long-term HFD.

To define the major genes/pathways that undergo post-transcriptional regulations in mild fatty liver, we performed GO term analysis for both our intron and exon DEG datasets (Supplementary Tables S2 and S3). As shown in Figure 3, genes with exon-level upregulation were enriched in the fatty acid metabolic process and organic acid biosynthetic process (Figure 3A), and genes with exon-level downregulation were mainly involved in fatty acid catabolism and the xenobiotic metabolic process in mild fatty liver (Figure 3B), which is consistent with our current knowledge about how mouse livers response to an HFD at the early stage [20,22]. While GO term analyses for genes with intron-level downregulation (Figure 3D) were similar to GO terms for genes with exon-level downregulation (Figure 3B), we found that GO terms for genes with intron-level upregulation were represented by synapse organization and cell adhesion (Figure 3C), which is not observed in the top GO terms for genes with exon-level upregulation in mild fatty liver (Figure 3A). These results are consistent with our correlation analysis as shown in Figure 2A. It also suggests genes involved in synapse organization and cell adhesion were post-transcriptionally suppressed, and this is the major factor contributing to the lower correlation between gene transcription and mature mRNA levels at the early stage of fatty liver. We next performed the same GO term analysis in severe fatty liver. We found that at both the intron and exon levels, upregulated genes were highly enriched for GO terms related to immune activation, and downregulated genes were highly enriched for GO terms related to small-molecule catabolic process (Figure 4A–D). These results are in line with our correlation analysis as shown in Figure 2B and are also consistent with our knowledge that a long-term HFD induces dramatic inflammatory responses and suppressed fatty acid catabolism and drug metabolism [21]. Taken together, our GO term analyses are consistent with our correlation analysis. They also revealed that genes involved in synapse organization and cell adhesion were post-transcriptionally suppressed at the early stage of fatty liver.

### 2.3. Post-Transcriptionally Suppressed Genes in Mild Fatty Liver Have Long UTRs

**Figure 5:**
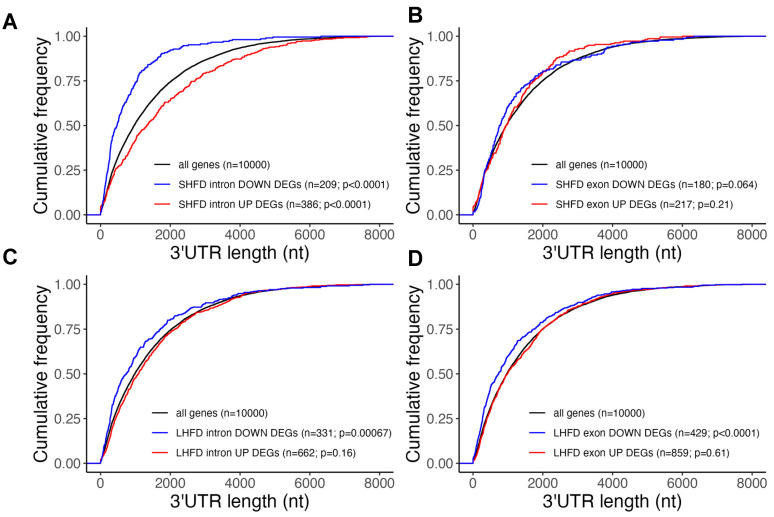
(**A**) 3′ UTR distribution for intron-level DEGs with short-term HFD. (**B**) 3′ UTR distribution for exon-level DEGs with short-term HFD. (**C**) 3′ UTR distribution for intron-level DEGs with long-term HFD. (**D**) 3′ UTR distribution for exon-level DEGs with long-term HFD.

**Figure 6:**
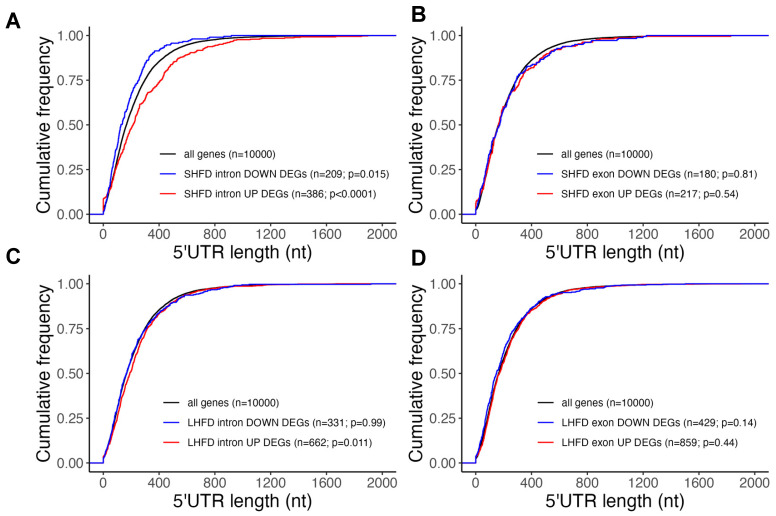
(**A**) 5′ UTR distribution for intron-level DEGs with short-term HFD. (**B**) 5′ UTR distribution for exon-level DEGs with short-term HFD. (**C**) 5′ UTR distribution for intron-level DEGs with long-term HFD. (**D**) 5′ UTR distribution for exon-level DEGs with long-term HFD.

To explore the potential mechanism underlying post-transcriptional regulations during fatty liver progression, we next asked if differentially expressed genes at early obesity shared any common features that may subject them to post-transcriptional regulations. Given the significance of the untranslated region (UTR) of mRNA in post-transcriptional regulations [23], we first systemically determined the 3′ UTR length distribution among the intron-/exon-level DEGs in both the short-term and long-term HFD settings. As shown in Figure 5A, we found that only genes with intron-level upregulation with the short-term HFD showed a significantly longer 3′ UTR when compared with the average UTR length of all liver-expressed genes or DEGs in other conditions. This result suggests that the long 3′ UTR may explain post-transcriptional suppression of many intron-level upregulated genes with the short-term HFD. We also found that genes with intron-/exon-level downregulation with either the short- or long-term HFD showed a shorter 3′ UTR when compared with the average UTR length of all liver-expressed genes (d), suggesting these genes may be subject to a lower level of post-transcriptional regulation. This is in line with our observation that GO terms for downregulated intron/exon DEGs with the short- and long-term HFD were similar. We further determined the 5′ UTR distribution in the same setting (Figure 6) and found that only genes with intron-level upregulation with the short-term HFD showed a significantly longer 5′ UTR when compared with the average UTR length of all liver-expressed genes (Figure 6A).

### 2.4. Post-Transcriptionally Suppressed Genes in Early Fatty Liver Are NMD Targets

**Figure 7:**
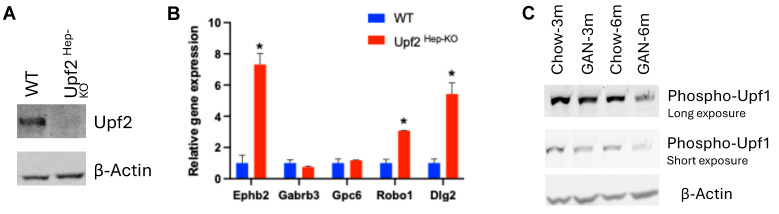
(**A**) Western blot analysis detecting Upf2 and β-Actin in the liver of WT or Upf2 ^Hep-KO^ mice. (**B**) qPCR detecting the relative expression of genes indicated (*n* = 4 for WT and *n* = 2 for Upf2 ^Hep-KO^). Data shown as the mean ± SEM, * *p* < 0.05. (**C**) Western blot analysis detecting phospho-Upf1 (Ser1127) or β-Actin in mice fed with chow or GAN diet for 3 or 6 months. Each lane represents samples pooled from 5 to 7 mice.

Having identified a long 3′ UTR as a major feature of post-transcriptionally suppressed genes in early fatty liver, we next searched for post-transcriptional mechanisms that were reported to correlate with 3′ UTR length. We noticed that nonsense-mediated RNA decay (NMD), an RNA quality control mechanism, not only targets mRNA with a pre-mature stop code but also shows a strong preference to degrade RNA transcripts with long 3′ UTRs [24,25,26]. To test if post-transcriptionally suppressed genes in early fatty liver are subjected to NMD, we prepared mice with hepatocyte-specific knockout of Upf2, a core component of NMD, by applying adeno-associated virus expressing Cre recombinase driven by the thyroxine binding globulin (TBG) promoter (AAV8-TBG-Cre) in Upf2^flox/flox^ mice [27,28,29]. As shown in Figure 7A, three weeks after AAV8-TBG-Cre injection, this strategy successfully depleted Upf2 protein expression in the liver. We next determined the expression of several representative genes, including Robo1, Gabrb3, Gpc6, Ephb2 and Dlg2, based on our analyses that (1) their expressions were post-transcriptionally suppressed in the short-term HFD and upregulated at both the intron and exon levels in the long-term HFD (Supplementary Figure S1); (2) they were involved in synapse organization and the cell adhesion process based on our GO term analysis and (3) they had extremely long 3′ UTRs. As shown in Figure 7B, we found that Upf2 knockout resulted in strong upregulation of Robo1, Ephb2 and Dlg2, suggesting they may be subjected to NMD. Given that the broad post-transcriptional regulations were mainly observed at the early stage of fatty liver, but not in severe fatty liver, we next asked if hepatic NMD activity is compromised during fatty liver progression. We thus measured the phosphorylation of Upf1, which is commonly used as an estimate of NMD activity [30], in the liver of mice fed with Gubra-Amylin NASH (GAN) diet [31] for 3 months or 6 months. As shown in Figure 7C, we found Upf1 phosphorylation was gradually suppressed upon GAN diet feeding with a substantial decrease at 6 months, when NASH-like phenotypes developed in these mice.

## 3. Discussion

Mammalian cells utilize multi-level regulatory mechanisms to control gene expression. At the mRNA level, gene expression is controlled by both transcription and degradation. In this study, we employed an RNA-seq data analysis pipeline that determines gene expression at both the intron and exon levels to study how gene expression in the liver adapts to high-fat diet feeding. Our major findings include the following: (1) in the early stage of HFD-induced fatty liver, there are broad post-transcriptional regulations to suppress genes involved in cell adhesion and synapse organization; (2) at the late stage of HFD-induced fatty liver, gene expression is mainly controlled by transcription; (3) post-transcriptionally suppressed genes in early fatty liver have extremely long 3-prime UTRs and other features including a longer 5-prime UTR and longer introns, making them more susceptible to post-transcriptional regulation; and (4) we provide evidence that post-transcriptionally suppressed genes in early fatty liver are subject to NMD, and suppressed NMD activity may partially explain the loss of post-transcriptional regulations in severe fatty liver. Our work thus provides an example of using the intron/exon mapping method in data analysis and its potential advances in hypothesis generation. Given that only exon-mapped reads were used for most of the published RNA-seq datasets, our strategy provides the advantage of deep mining RNA-seq datasets to maximize the usage of the sequencing data.

It is well known that upon stress/stimulations, cells adapt by controlling gene expression/activity at multiple layers. Our study here revealed that upon HFD feeding, mice utilize both transcriptional and post-transcriptional mechanisms to maintain metabolic homeostasis. Specifically, we found that genes enriched for synapse organization and cell adhesion were post-transcriptionally suppressed at the early stage of fatty liver. These genes, including Robo1, Gabrb3, Gpc6, Ephb2 and Dlg2, are usually not expressed in healthy liver, and were reported to be upregulated in metabolic dysfunction-associated steatohepatitis and liver fibrosis. In particular, increased Robo1 and Ephb2 expression has been experimentally proven to promote MASH and liver fibrosis [32,33]. Our results suggest that post-transcriptional regulations represent a checkpoint to suppress the expression of unwanted/harmful genes in the liver upon HFD-induced nutritional stress. In our setting, short-term HFD feeding (7–8 weeks) very likely represents a turning point, after which metabolic disease phenotypes may develop. Indeed, it is well accepted that with increased body weight and mild fatty liver, mice fed with an HFD for 8 weeks show no significant metabolic defects [34].

The finding that post-transcriptionally regulated genes upon HFD feeding in the liver show features, including a long 3′ UTR, suggests that they may be subject to NMD regulation. Given many of these genes are induced in disease states and promote liver disease progression, their expression is thus under tight surveillance by mechanisms such as NMD. Indeed, for genes that are mainly controlled by transcriptional regulations, such genes with exon-level downregulation at the early stage of fatty liver have a significantly shorter 3′ UTR as compared with all liver-expressed genes. These findings suggest that 3′ UTR length alone may determine to what extent a gene’s expression may be subject to post-transcriptional regulations such as NMD. Our result that Upf1 phosphorylation was maintained at the early stage of fatty liver but lost in severe fatty liver supports the conclusion that loss of NMD activity may explain low post-transcriptional regulations in severe fatty liver diseases and possibly other chronic liver diseases including MASH, liver fibrosis and liver cancer. It was reported that NMD activity can be suppressed by many environmental factors, including ER stress, a known feature of diet-induced fatty liver [35,36]. Our observation also suggests that restoring NMD activity may show a protective effect in liver diseases by suppressing the expression of disease-related genes, such as Robo1 and Ephb2.

The purpose of this study is to use RNA-seq reads mapped to all intron regions of a gene as an estimate of pre-mRNA expression and use RNA-seq reads mapped to all exon regions of a gene as an estimate of mature mRNA. By comparing how gene expression changes at the pre-mRNA level correlate with those at the mature mRNA level, we aim to predict the contribution of post-transcriptional regulations. As such, the design of the pipeline is not able to measure alternative splicing events. Indeed, to avoid the potential confounding effects of alternative splicing events, including intron retention, we only use reads that map to constitutive exons (exons share by all reported transcript isoforms of a gene) for determining exon DEGs, and only use reads that map to constitutive introns (introns share by all reported transcript isoforms of a gene) for determining intron DEGs. The transcript isoform annotation is based on the GRCm39 Ensemble mouse genome. It is possible that unannotated alternative splicing events, including intron retention and alternative splicing of the 3′ UTR, may affect our intron DEG analysis. Given the nature of our RNA-seq analysis pipeline, we suggest only using published RNA-seq datasets with libraries prepared by total RNA (18S depleted) and with total reads more than 50 M for each sample, to ensure enough intron-mapped reads for analysis. Other limitations associated with the two datasets include the age difference of mice, mouse species (C57BL/6J in GSE88818 versus C57BL/6N in GSE121340), diet compositions (40% fat in GSE88818 versus 60% fat in GSE121340), distinct RNA sequencing techniques, etc., which could all serve as confounding factors that potentially impact our conclusions. Future studies using datasets representing the early and late stages of fatty liver in mice with the same genetic background, same HFD and same RNA-seq pipeline will be ideal to exclude the confounding factors.

## 4. Materials and Methods

### 4.1. Reanalysis of Publicly Available RNA-Seq Data

We downloaded 6 samples from dataset GSE88818, obtained from 19-week-old control mice (*n* = 3) fed a chow diet and 19-week-old obese mice (*n* = 3) fed an HFD (D12327, Research Diet, 40% fat by calories). HFD feeding started at 12 weeks of age and continued for 7 weeks. We also downloaded 6 samples from dataset GSE121340, obtained from 36-week-old control mice (*n* = 3) fed a chow diet and 36-week-old obese mice (*n* = 3) fed an HFD (D12492, Research Diet, 60% fat by calories). HFD feeding started at 6–7 weeks of age and continued for 29–30 weeks. The Fastq files were downloaded and prepared using the SRA Toolkit (version 2.11.1) (https://www.ncbi.nlm.nih.gov/books/NBK569238/, accessed on 8 September 2024). Briefly, the prefetch command was used to download the SRA files of the dataset and convert them into FASTQ format using the fasterq-dump command. The quality of the Fastq files was evaluated using FastQC (version 0.11.8) (http://www.bioinformatics.babraham.ac.uk/projects/fastqc/, accessed on 8 September 2024), and adapters and low-quality reads were trimmed or removed using Trimmomatic (version 0.39) [37]. The filtered reads were then mapped to the GRCm 39 Ensembl mouse genome using STAR (version 2.7.8a) [38]. Annotation files for gene regions to obtain mature mRNA and pre-mRNA count data were created as follows. From the annotation file of the mouse genome, transcripts whose transcript source was ensembl or ensembl_havana were extracted. From there, exon regions common to all transcripts for each gene were extracted and designated as constitutive exon regions. For the creation of the intron region definition file, the region between adjacent exons was extracted for each gene. Gene-level counts for mature mRNA and pre-mRNA were generated using featureCounts from Subread (version 2.0.0) [39] by using uniquely mapped reads in the union exon region or the union intron region, respectively. In each dataset, genes with two or fewer samples with raw counts greater than one were pre-filtered out. The pre-filtered raw count matrix was normalized by DESeq2 (version 1.36.0) [40] on R (version 4.1.0). Differentially expressed genes were defined as genes with a false discovery rate (FDR) ≤ 0.05 and |Log2FC| ≥ 0.58. DEGs in the top 10,000 in baseMean calculated by DESeq2 were used as an input for gene ontology (GO) enrichment analysis using clusterProfiler (version 4.0.5) [41]. In the GO enrichment analysis, GO terms with an adjusted *p*-value ≤ 0.01 were considered significant.

### 4.2. UTR Feature Analysis

Transcript IDs of the Ensembl Canonical Transcripts of mice were collected using biomaRt (version 2.58.2) [42] as representative transcripts for each gene. Based on the list of Transcript IDs, annotation information such as 3′UTR and 5′UTR length was generated using biomaRt, GenomicRange (version 1.54.1) [43], AnnotationHub (version 1.64.1) [DOI: 10.18129/B9.bioc.AnnotationHub] and Biostrings (version 2.70.3) [DOI: 10.18129/B9.bioc.Biostrings] on R (version 4.1.0). The distribution of UTR lengths for the top 10,000 genes in the baseMean of each dataset was compared to the distribution of UTR lengths for DEGs within the top 10,000 genes, and statistical significance was assessed with a two-tailed Kolmogorov–Smirnov test.

### 4.3. Mouse Study

Upf2^flox/flox^ mice were as previously described [44]. AAV8-TBG-Cre and AAV8-CMV-GFP controls were purchased from Vector Biolabs. Six-week-old male Upf2^flox/flox^ mice were injected with 2 × 10^11^ genome copies/mouse. Three weeks after AAV injection, liver tissues were harvested for RNA and protein extraction. For the GAN diet study, 8-week-old C57BL/6J mice were fed with normal chow or the GAN diet (Research Diets, D09100310) for 3 months or 6 months, and then the liver tissue was harvested for Western blot analysis. Mouse experiments were approved by the Johns Hopkins University Animal Care and Use Committee (ACUC).

### 4.4. RNA Extraction and qPCR

Total RNA was extracted and purified using the Qiagen RNeasy Mini Kit (Qiagen Cat# 74106, Hilden, Germany) following the manufacturer’s instructions and performing on-column DNA digestion. An amount of 200–500 ng of total RNA was used for cDNA synthesis with the SuperScript III First-Strand Synthesis SuperMix for qRT-PCR (Invitrogen Cat# 11752050, Waltham, MA, USA). cDNA was diluted 3–5-fold with RNase-free water. qPCR reactions consisted of 7.5 µL of the Power SYBR Green PCR Master Mix (ThermoFisher Cat# 4368702, Waltham, MA USA), 1.5 μL of cDNA and 6 µL of 0.5 µM primers (primers are listed in Table 1). qPCR was performed using a ThermoFisher Quantstudio 7 Flex using a 386-well plate (ThermoFisher Cat# 4309849) in Standard Mode. Target gene expression was normalized by the ΔΔCT method and 18S was used as the internal control. The qPCR primers used are listed below.

### 4.5. Protein Extraction and Western Blotting

Total protein was extracted using 1× LDS loading buffer (1× NuPAGE LDS Sample Buffer (Invitrogen Cat# NP0007), 0.5% β-mercaptoethanol, 1 mM PMSF and EDTA-free Protease Inhibitor Cocktail (PI78439). Protein lysates were boiled at 70 °C for 10 min and stored at −20 °C until used. Samples were pre-heated at 37 °C for 5 min and loaded to 4–12% NuPAGE™ Bis-Tris Mini Protein Gels (Invitrogen Cat# NP0322PK2) set in XCell SureLock Mini-Cell (Invitrogen Cat# EI0001) filled with 1× NuPAGE MOPS SDS Running Buffer (Invitrogen Cat# NP000102) and the electrophoresis was carried out using a PowerPac Basic Power Supply (Bio-Rad Cat# 1645050, Hercules, CA, USA). Proteins in the gel were transferred onto 0.45 μm Immobilon-FL transfer membranes (Millipore Cat# IPFL00010, Burlington, MA, USA) using an XCell II Blot Module (Invitrogen Cat# EI9051) filled with 1× NuPAG Transfer Buffer (Invitrogen Cat# NP00061) with 0.01% SDS and 5% methanol. After membranes were blocked with Intercept (PBS) Blocking Buffer (LI-COR Cat# 927-70001, Nebraska, NE, USA), proteins of interest were probed with specific primary antibodies and then appropriate Fluorescent Dye-conjugated secondary antibodies diluted in Intercept T20 (TBS) Antibody Diluent (LI-COR Cat# 927-75001). Fluorescent signals were detected using a ChemiDoc MP Imaging System (Bio-Rad Cat# 12003154). The following primary antibodies were used for immunoblotting: Upf2 (Cell Signaling Technology, Cat# 11875, Danvers, MA, USA), β-Actin (Cell Signaling Cat# 8457), phospho-Upf1 (Ser1127) antibody (MilliporeSigma, 07-1016, Burlington, MA, USA). The following secondary antibodies were used: IRDye 800CW Goat anti-Rabbit IgG Secondary Antibody (LI-COR Cat# 926-32211) and IRDye 800CW Goat anti-Mouse IgG Secondary Antibody (LI-COR Cat# 926-32210).

## Figures and Tables

**Table 1:** Primer list.

Primer Name	Sequence
18S-f	AGTCCCTGCCCTTTGTACACA
18S-r	CGATCCGAGGGCCTCACTA
Ephb2-f	AGGCAAGCAACAAGGAAAGG
Ephb2-r	GCAGGGCCCAGAGTATATGT
Gabrb3-f	ATAAACCGGGTGGATGCTCA
Gabrb3-r	TGGGCATGCTCTGTTTCCTA
Gpc6-f	AAGCCAGATACCTGCCTGAG
Gpc6-r	TGTTGGTCATCACACGGAGA
Robo1-f	GCTGCCAAGCGGGTCTTTAT
Robo1-r	CTCCGAGGTAATTCCTAGCCA
Dlg2-f	GTGAGATTTGTAGCAGAAAGGGG
Dlg2-r	GGCGACTTGTAACCGCTTAATAG

## Data Availability

The original contributions presented in the study are included in the article, further inquiries can be directed to the corresponding author.

## Supplementary Materials

The following supporting information can be downloaded at: https://www.mdpi.com/article/10.3390/ijms252011054/s1.
