# Supplementary material for: Disrupted Post-Transcriptional Regulation of Gene Expression as a Hallmark of Fatty Liver Progression
Source: Int J Mol Sci. 2024 Oct 15;25(20):11054. doi: 10.3390/ijms252011054 (PMC11507451; doi:10.3390/ijms252011054)
Supplement: Supplementary file 1 [file ijms-25-11054-s001.zip › Supplementary Figure S1.pdf]

Supplementary Figure S1

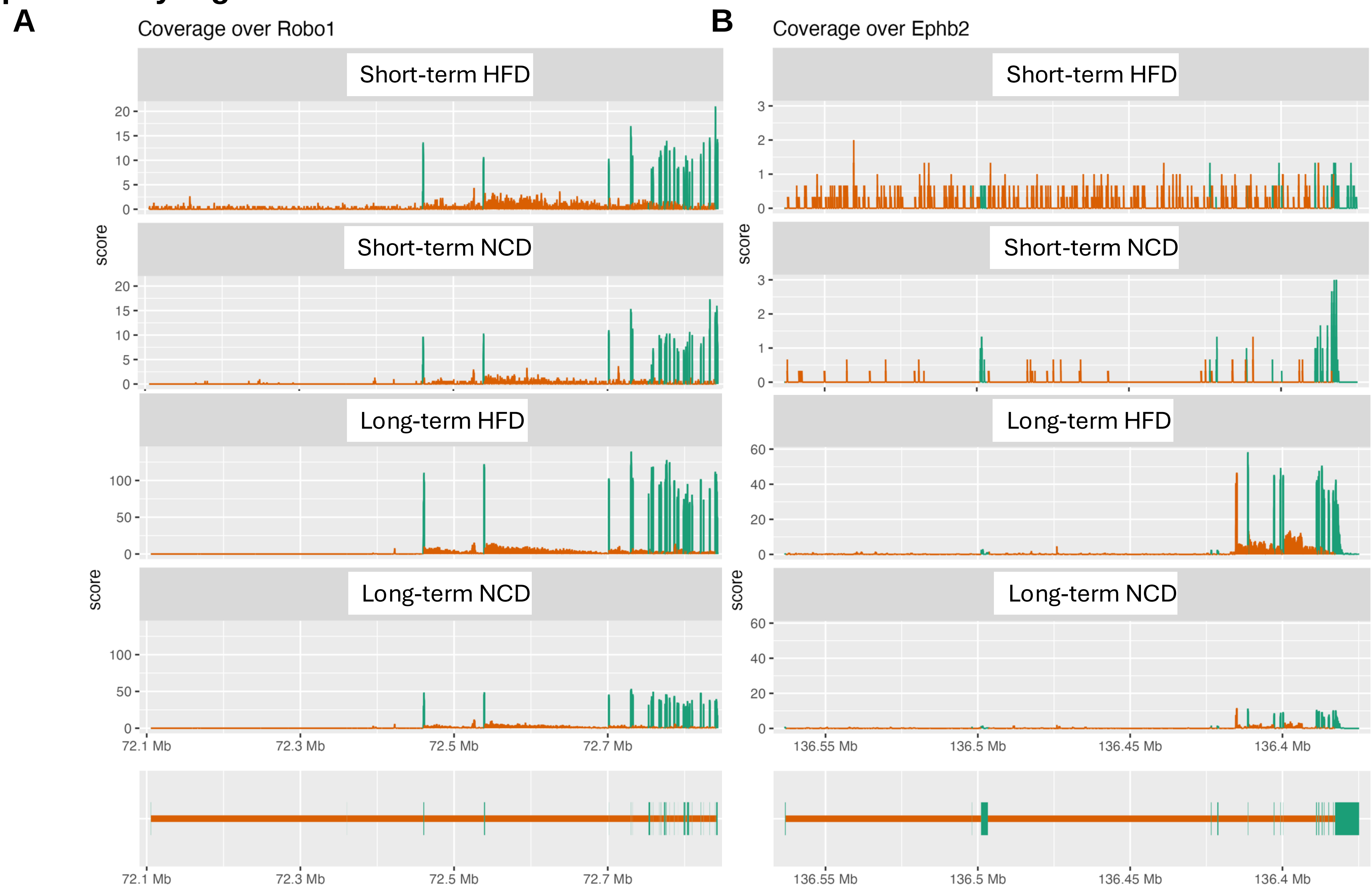

Supplementary Figure S1. RNA-seq coverage plots of potential NMD target genes. RNA-seq read coverage over (**A**) Robo1, and (**B**) Ephb2 genes were analyzed using the Superintronic. Coverage scores are plotted on the y-axis, while gene coordinates are shown on the x-axis. The green regions correspond to exons, and the orange regions represent introns. A gene structure diagram is included at the bottom of each panel, indicating the positions of exonic and intronic regions within each gene and providing a reference for the corresponding RNA-seq read coverage. NCD, normal chow diet.
